# Keep it simple: streamlining book illustrations improves attention and comprehension in beginning readers

**DOI:** 10.1038/s41539-020-00073-5

**Published:** 2020-09-28

**Authors:** Cassondra M. Eng, Karrie E. Godwin, Anna V. Fisher

**Affiliations:** 1 Department of Psychology, Carnegie Mellon University, 346 Baker Hall, 5000 Forbes Avenue, Pittsburgh, PA 15213 USA; 2 Department of Psychology, University of Maryland Baltimore County, Baltimore, USA

**Keywords:** Education, Human behaviour, Human behaviour

## Abstract

This study used eye-tracking to examine whether extraneous illustration details—a common design in beginning reader storybooks—promote attentional competition and hinder learning. The study used a within-subject design with first- and second-grade children. Children (*n* = 60) read a story in a commercially available Standard condition and in a Streamlined condition, in which extraneous illustrations were removed while an eye-tracker recorded children’s gaze shifts away from the text, fixations to extraneous illustrations, and fixations to relevant illustrations. Extraneous illustrations promoted attentional competition and hindered reading comprehension: children made more gaze shifts away from text in the Standard compared to the Streamlined condition, and reading comprehension was significantly higher in the Streamlined condition compared to the Standard condition. Importantly, fixations toward extraneous details accounted for the unique variance in reading comprehension controlling for reading proficiency and attending to relevant illustrations. Furthermore, a follow-up control experiment (*n* = 60) revealed that these effects did not solely stem from enhanced text saliency in the Streamlined condition and reproduced the finding of a negative relationship between fixations to extraneous details and reading comprehension. This study provides evidence that the design of reading materials can be optimized to promote literacy development in young children.

## Introduction

Learning to read is a crucially important skill because reading provides a gateway for learning within and outside of school. However, many children struggle to acquire the fundamental skill of learning to read and one-third of U.S. elementary school students are not reading at grade level^1^. Many factors contribute to children’s difficulty in learning to read, including (but not limited) to: neurodevelopmental disorders, lagging pre-reading skills (e.g., phonological awareness), and vulnerabilities in general cognitive functioning^2–4^. This study focuses on one potential factor that has received relatively little attention in the literature, namely the design of reading materials for beginning readers.

The typical design of books for beginning readers often includes engaging, colorful, detailed illustrations. There are a number of reasons for including illustrations in books for beginning readers such as defining the setting and characters, contributing to text coherence, reinforcing the text, providing additional information, and motivating the reader^5,6^. Yet, attention is a competitive process and only a subset of information can be selected for processing and represented in visual working memory^7–9^. In beginning readers, for whom reading has not yet become an automatized skill, engaging illustrations may compete for attention with text. If illustrations and text indeed compete for children’s attention, then the inclusion of extraneous illustrations may undermine children’s reading comprehension. Looking away from the text at illustrations may result in the encoding of irrelevant details into a working memory which may ultimately disrupt text coherence. It may be difficult for beginning readers to build a strong understanding of the story if they attend to extraneous illustrations while reading. Furthermore, attention regulation skills are still developing during the time when children begin formal reading instruction^10–12^; therefore, it is important to evaluate the possibility that unnecessary embellishments to educational materials intended to engage children might do so at the cost of disrupting attention and learning^13–16^. This possibility is particularly important to evaluate in light of the evidence that individual differences in selective attention are related to individual differences in reading skills^17–20^. Entertaining visuals in children’s educational materials have enormous potential to engage children—but these additional visuals might be counterproductive if they are unrelated to the story text as they may distract children from the primary task (i.e., decoding the words and making meaning from the text).

Extraneous details—also known as seductive details—are often included to increase motivation and foster situational interest^21^. There is a substantial body of research on the detrimental effects of extraneous details in educational materials with adult populations. The inclusion of extraneous details has been found to hinder the ability to recall important ideas and comprehension of material in scientific texts^22^, in lectures^23^, and in online lessons^24^. The Cognitive Load Theory suggests that unnecessary or extraneous material reduces the number of cognitive resources available for the target task and decreases performance and learning outcomes^25–28^. Multimedia design principles based on the Cognitive Load Theory suggest that when learners have to divide attention between images and text (Split-Attention Principle) and process irrelevant information (Coherence Principle) comprehension is significantly reduced^29,30^. In contrast to the large body of research on the design of educational materials for adult learners who are reading-to-learn, relatively few studies have examined this issue in children who are learning-to-read.

There is evidence that specific attributes of picture books influence the experiences of shared book reading for pre-reading children^31,32^. For example, Flack and Horst^31^ found in shared reading contexts with pre-readers that the presence of multiple illustrations per page was detrimental for children’s word learning^31^. A handful of studies with beginning readers suggests that presenting text without illustrations results in faster reading time and higher accuracy in elementary school students^33–35^. Evidence on the effects of illustrations on reading comprehension is mixed. Some studies suggest beneficial effects of including illustrations in text for beginning readers. For example, Rusted and Coltheart^36^ constructed prose passages describing novel creatures and asked 9 to 13-year-old children to read the passages either accompanied by a simple line drawing of a novel creature or without the drawing^36^. The results indicated that including simple line drawings with the passages improved recall of passage details in both good and poor readers. Hannus and Hyönä^37^ also reported positive effects of black-and-white line drawings on 4th-grade students’ comprehension of passages in a biology textbook (compared to a no-illustration condition); although, this effect was observed for students who scored high on a separately administered test of non-verbal intelligence and not for students who scored low on this test^37^. Other studies point to the detrimental effects of illustrations in beginning reader materials, particularly for struggling readers and children with learning disabilities. Rose^38^ conducted one of the few studies that used ecologically valid materials designed for independent reading practice and found the comprehension scores of students with learning disabilities were significantly higher for non-illustrated than for illustrated passages^38^. Coldstein and Underwood^39^ reviewed different styles of illustrations in reading materials for beginning readers and noted, “Clearly a variety of assumptions have been made by designers of reading scheme, but studies of the processes involved in learning to read have shed very little light on the role of illustration,” (p. 9)^39^. Nearly four decades later, we still know relatively little about the role of illustrations in reading materials for beginning readers and a number of questions remain unresolved.

First, several researchers have suggested that pictures may distract children from printed text^38–40^. Some researchers have further proposed that when text is accompanied by illustrations, working memory resources are devoted to processing pictures; thus, less resources are left for processing written text^24,34^. Yet, there is no direct evidence that beginning readers are distracted by illustrations, or that children attend less to the text in the presence of illustrations. Eye-tracking studies of shared story-book reading in pre-reading children suggest that pre-reading children overwhelmingly attend to images and only minimally attend to text^41,42^, whereas eye-tracking evidence from studies with older children who are reading-to-learn suggests that by fourth-grade children overwhelmingly attend to text and only minimally to illustrations^37^. However, no prior studies used eye tracking to examine the effect of illustrations on reading performance in beginning readers. Beginning readers are in a transition period: during primary school years children still experience social reading contexts such as a shared book reading with a teacher or caregiver, but beginning readers are also increasingly expected to read independently. The present work is aimed at understanding how best to support young readers’ during this transition period as they become independent fluent readers. Second, most prior studies focused on decoding—children’s ability to read words quickly and accurately—but few studies examined the effects of illustrations on reading comprehension. It is possible the detrimental effect of illustrations on decoding may be offset by the beneficial effects of illustrations on reading comprehension^36^. Indeed, instructing children to refer to illustrations to aid comprehension as well as decoding is a common instructional strategy in elementary school^40,43^. Alternatively, it is possible that by interfering with decoding, illustrations may also interfere with reading comprehension, as Torcasio and Sweller^34^ suggested, and as Rose^38^ observed in students with learning disabilities^34,38^. Lastly, prior research has investigated illustrations in materials for beginning readers in a binary fashion: illustrations were either present or absent. Perhaps the putative negative effects of illustrations on children’s reading performance can be minimized simply by removing extraneous illustrations, rather than by completely removing illustrations from beginning reader books.

**Fig. 1:**
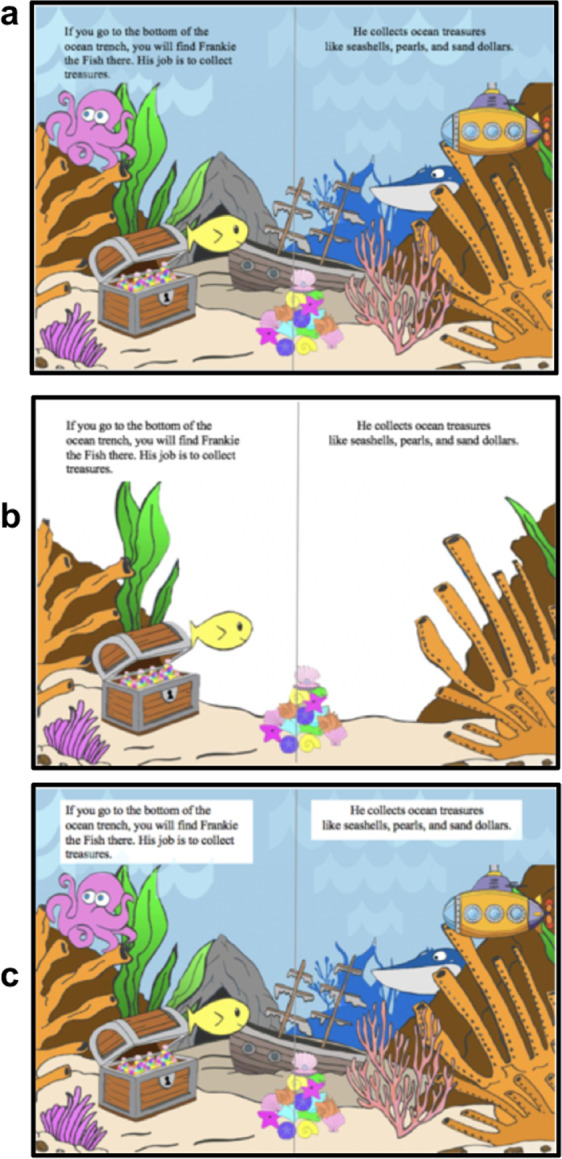
Reading materials schematic by condition. Schematic depiction of a book page in the Standard condition (top) in which the illustrations include extraneous details; the Streamlined condition (middle) in which the extraneous details were removed and only illustrations relevant to the text were retained; and the Featureless Background condition (bottom) in which the illustrations were identical to the Standard condition, however, the text was placed on a plain featureless background. Note that these are original images hand-drawn and developed by the first author of the study to schematically represent the differences among conditions. Actual images of the reading materials used in the study are not reproduced here to avoid copyright infringement. Examples of the reading materials and sample gaze patterns of children reading in each condition can be viewed in the Open Science Framework repository for the study: https://osf.io/frgw8/?view_only=42259f9134024b54bd5adae2da7f9c2a.

This study aimed to address the limitations above. We examined the effects of extraneous illustrations on attention and reading comprehension in 1st- and 2nd-grade students (*n* = 60) using a commercially available book designed for independent reading practice in 1st grade. Half of the book was presented to children in a commercially available “Standard” condition, and half of the book was presented to children in a “Streamlined” condition in which extraneous illustrations were removed (schematic depiction of reading materials in each condition is shown in Fig. 1). The order of conditions was counterbalanced across participants and presented to children on a computer screen while an SMI RED250 mobile eye tracker recorded children’s eye gaze patterns. The primary measures of children’s attention during reading were gaze shifts away from text and fixations to extraneous details. A calibration study with adult fluent readers was conducted to methodically determine which illustrations were extraneous (see Method). Comprehension questions provided by the book publisher were administered to assess reading comprehension and were linked to the content presented on specific pages, making it possible to clearly distinguish events from the first or second half of the book (see “Methods” for sample questions). To control for reading level, reading accuracy was assessed using a Running Record^44^, which measures the percentage of words children decode accurately aloud. An independent assessment of reading proficiency—Word Recognition in Isolation test (WRI)^45^—was also administered.

Based on the theoretical framework of attention as a competitive process^7–9^, we expected that if text and illustrations compete for children’s attention, there should be a higher rate of gaze shifts away from the text in the Standard condition compared to the Streamlined condition. In line with the prediction of the Cognitive Load Theory^30,34^, we hypothesized that encoding extraneous illustrations in the Standard condition would negatively affect children’s reading comprehension compared to the Streamlined condition. To rule out the possibility the observed effects in the Streamlined condition stemmed from enhancing text saliency, we conducted a follow-up control experiment with another sample of 1^st^ and 2nd-grade students (*n* = 60) implementing a Featureless Background condition (see Fig. 1 for a schematic depiction of reading materials in this condition).

## Results

### Reading level

Children were beginning readers as evidenced by their performance on the WRI, the independent measure of children’s reading proficiency (*M* = 68.87, SD = 18.89). The selected book was an appropriate difficulty level for independent reading based on children’s mean performance on the Running Record (*M* = 96.56%; SD = 4.15%). The manipulation to the book condition did not influence children’s decoding accuracy (Standard: *M* = 95.79%; SD = 4.13%; Streamlined: *M* = 95.78%; SD = 4.16%), paired-sample *t*(59) = 0.89, *p* = 0.38; Cohen’s *d* = 0.12.

### Eye-tracking results

Eye-tracking data from 1 participant were not included in the analyses due to a technical failure. There were no significant differences in total looking duration at the book pages in the Standard condition (*M* = 42,339 ms; SD = 30,459 ms) compared to the Streamlined condition (*M* = 40,325 ms; SD = 31,856 ms), paired-sample *t*(58) = 1.09, *p* = 0.28; Cohen’s *d* = 0.14.

#### Gaze shifts

**Fig. 2:**
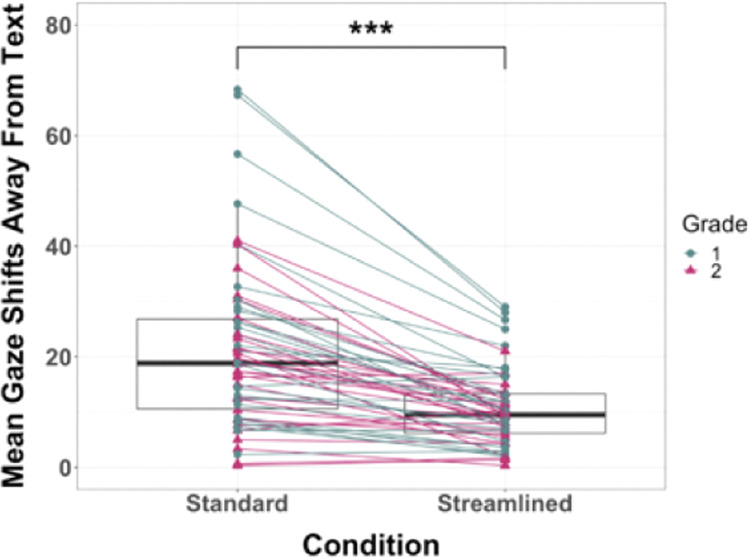
Mean gaze shifts by condition. Paired box plot of mean gaze shifts away from the text in the Standard and Streamlined book conditions. Children looked away from the text more frequently while reading in the Standard condition compared to the Streamlined condition. This pattern was observed for both first and second-grade children; although a trend was observed in which first-grade children (teal markers) looked away from the text more than second-grade children (pink markers). Boxplot center line identifies the median, the upper whiskers extend from the 75th percentile to the 75th percentile + 1.5 interquartile range, the lower whiskers extend from the 25th percentile to the 25th–1.5 interquartile range, ***p < 0.001. Note: One extreme outlier (gaze shift value of 134.33 in the Standard condition) is not displayed.

First, to examine the effect of removing extraneous illustrations on attention, we assessed how frequently children looked away from the text in each book condition. To assess possible order effects and grade differences, we conducted a linear mixed-effects model (LMM) on gaze shifts away from the text, with book condition, grade, and order modeled as fixed effects and subject as a random effect. Table 1 shows the estimations of fixed effects and the corresponding 95% confidence intervals (CI). There was a main effect of book condition, *F*(1, 58) = 40.26; *p* < 0.0005; Cohen’s *d* = 0.83. The fixed intercept value of 4.54 represents the mean gaze shifts away from the text while reading in the Streamlined condition. The intercept for gaze shifts away from the text in the Standard condition is 4.54 + 12.31 = 16.85, and this is significantly higher than the mean gaze shifts away from the text while reading in the Streamlined condition (*t* = 6.35, *p* < 0.0005, 95% CI for the difference is 8.42 to 16.19 higher). Follow-up pairwise comparisons after Bonferroni corrections revealed that on average, children looked away from the text 12.31 (SE = 1.94) more times per page in the Standard condition compared to the Streamlined condition. There was one extreme outlier (i.e., average gaze shifts that deviated >3 SD away from the group mean) in the Standard condition. With the removal of this outlier, there was still evidence of a main effect of book condition on gaze shifts away from the text, *F* = 39.53; *p* < 0.0005; Cohen’s *d* = 0.89. There was no significant main effect of grade with alpha set at 0.05, *F*(1, 56) = 3.78, *p* = 0.057; Cohen’s *d* = 0.42. However, the effect size is medium, indicating a trend of first-grade children looking away from the text while reading more than second-grade children: the intercept for gaze shifts away from the text for first-grade children is 4.54 + 6.99 = 11.53, and this is moderately higher than the mean gaze shifts away from the text for second-grade children (*t* = 1.95, *p* = 0.057, 95% CI for the difference is −0.21 to 14.21 higher). There was no main effect of order, *F*(1, 56) = 1.91, *p* = 0.173; Cohen’s *d* = 0.29, and no significant interactions between any of these factors and gaze shifts away from the text (all *p*s > 0.17; see Table S5 in the online Supplementary Material for LMM analysis with the interaction terms). Taken together, these results support the prediction that children look away from the text at a higher rate while reading the story in the Standard condition than in the Streamlined condition (see Fig. 2 for paired box plot).

**Table 1:** Estimates of fixed effects obtained using the linear mixed-effects model.

Outcome Variable	Parameter	Estimate	SE	df	*t*	*p*	95% CI
Gaze shifts away from text	Intercept	4.54	3.32	66.40	1.37	0.175	[−2.08, 11.16]
	[Condition = Standard]^a^	12.31	1.94	58.00	6.35	<0.0005	[8.42, 16.19]
	[Grade = 1]^b^	6.99	3.60	56.00	1.95	0.057	[−.210, 14.21]
	[Order = Standard First]^c^	4.98	3.60	56.00	1.38	0.173	[−2.24, 12.19]
Comprehension scores (%)	Intercept	79.69	4.11	90.52	19.41	<0.0005	[71.53, 87.84]
	[Condition = Standard]^a^	−32.86	4.05	59.00	−8.11	<0.0005	[−40.96, −24.75]
	[Grade = 1]^b^	−3.48	4.12	57.00	−0.844	0.402	[−11.74, 4.78]
	[Order = Standard First]^c^	4.44	4.13	57.00	1.08	0.287	[−3.82, 12.70]

^a^The LMM model used results from Standard condition as the reference.

^b^The LMM model used results from Grade 1 as the reference.

^c^The LMM model used results from the order of the Standard condition presented first as the reference.

### Reading comprehension

**Fig. 3:**
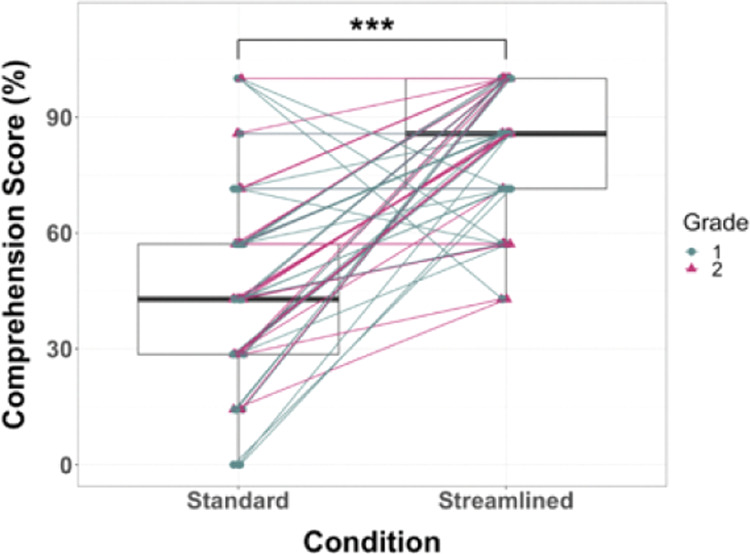
Reading Comprehension Accuracy by Condition. Paired box plots of reading comprehension scores (%) in the Standard and Streamlined conditions. Reading comprehension scores were higher in the Streamlined condition compared to the Standard condition. Boxplot center line identifies the median, the upper whiskers extend from the 75th percentile to the 75th percentile + 1.5 interquartile range, the lower whiskers extend from the 25th percentile to the 25th–1.5 interquartile range, ***p < 0.001. Data points were jittered in R by 0.03 to prevent overplotting (Team^67^). Note: One outlier (score of 28.57% in the Streamlined condition) is not displayed.

To investigate our primary hypothesis—that removing extraneous details would improve reading comprehension—we assessed how well children could answer questions related to the content of the story they read in each book condition. To assess possible order effects and grade differences, we conducted a LMM on reading comprehension, with book condition, grade, and order modeled as fixed effects and subject as a random effect. Table 1 shows the estimations of fixed effects and the corresponding 95% CIs. There was a main effect of book condition, *F*(1, 59) = 65.80; *p* < 0.0005; Cohen’s *d* = 1.05. The fixed intercept value of 79.69 represents the mean comprehension scores (in %) for the Streamlined condition. The intercept for comprehension scores in the Standard Condition is 79.69−32.86 = 46.83, and this is significantly lower than comprehension scores in the Streamlined condition (*t* = −8.11, *p* < 0.0005, 95% CI for the difference is 24.75–40.96% lower). Follow-up pairwise comparisons after Bonferroni corrections revealed that on average, children scored 32.86% (*SE* = 4.05) higher on the comprehension assessment in the Streamlined condition compared to the Standard condition. There was one outlier with a comprehension score of 28.57% in the Streamlined condition. With the removal of this outlier, there was still evidence of a main effect of book condition on comprehension, *F* = 69.91; *p* < 0.0005; Cohen’s *d* = 1.05. There was no main effect of order, *F*(1, 57) = 1.16, *p* = 0.287; Cohen’s *d* = 0.16, or grade, *F*(1, 57) = 0.71, *p* = 0.402; Cohen’s *d* = 0.12, and no significant interactions between any of these factors and comprehension (all *p*s > 0.28; see Table S5 in the online Supplementary Material for LMM analysis with the interaction terms). These findings support the prediction that reading comprehension scores would be higher in the Streamlined condition than in the Standard condition (see Fig. 3 for paired box plot).

### Association between eye gaze patterns and reading comprehension

**Fig. 4:**
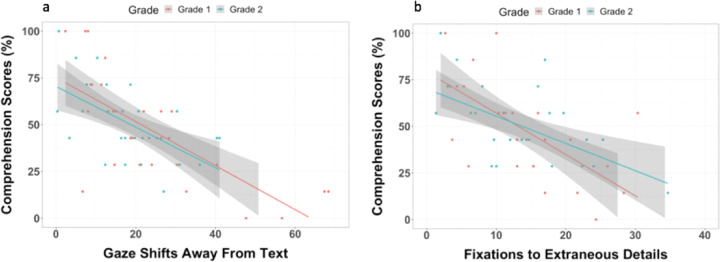
Scatterplots for association between reading comprehension and eye gaze patterns. Scatterplots of correlations between reading comprehension scores (% correct) and eye gaze patterns in the Standard condition. Higher gaze shifts away from the text (left) and fixations to extraneous details (right) were both negatively associated with reading comprehension scores. Shaded regions represent 95% confidence interval of the prediction line.

We then examined the association between mean gaze shifts away from the text and fixations to extraneous details while reading and reading comprehension performance. It is plausible children looked away from the text more in the Standard condition compared to the Streamlined condition because children may attempt to use the illustrations as a strategy to help determine the meaning of unknown words. However, increased gaze shifts away from the text, *r*(57) = −0.62, 95% CI [−0.75, −0.43], *p* < 0.0005, and higher fixations to extraneous details *r*(57) = −0.42, 95% CI [−0.61, −0.18], *p* < 0.0005, were negatively associated with children’s comprehension scores in the Standard condition (see Fig. 4). In other words, the associations between reading comprehension scores with children’s eye gaze patterns indicate that not only are gaze shifts away from the text negatively associated with children’s reading comprehension performance, but children who often fixate on extraneous illustrations while reading have lower reading comprehension scores.

**Fig. 5:**
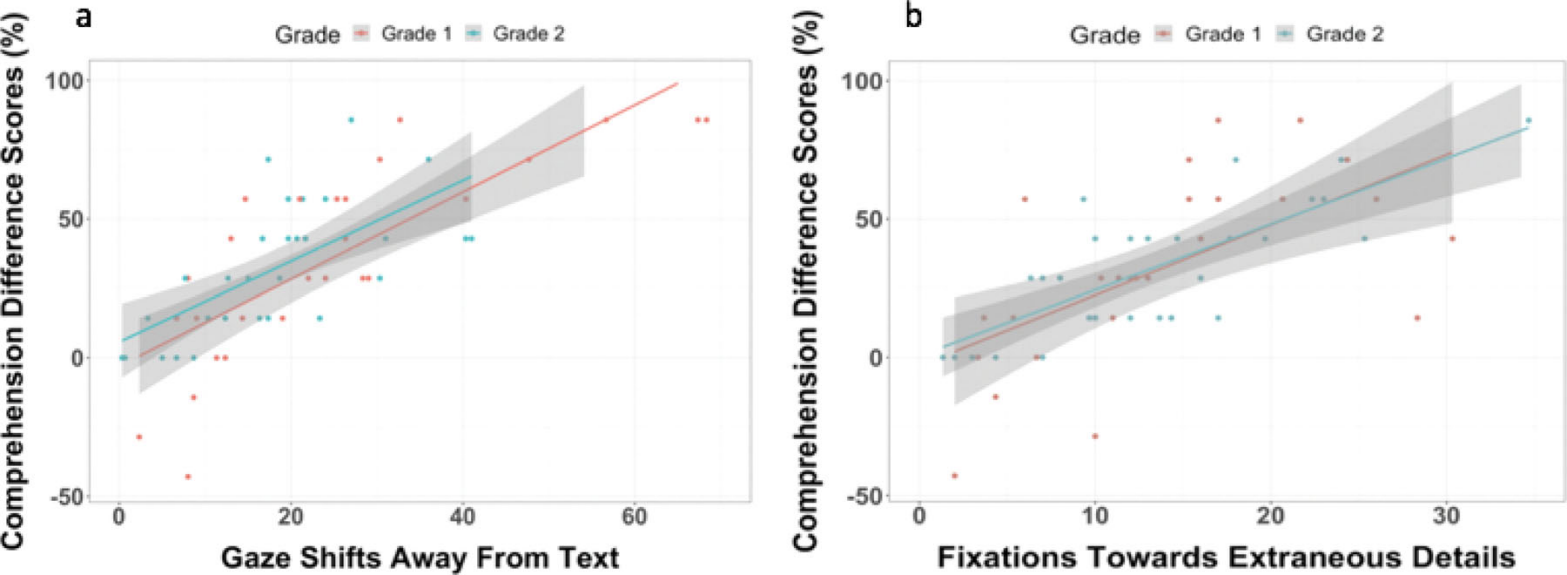
Scatterplots for association between comprehension difference scores and eye gaze patterns. Scatterplots of the correlations between reading comprehension difference scores (Streamlined Reading Comprehension Score–Standard Comprehension Score) and eye gaze patterns. Higher gaze shifts away from the text (left) and fixations to extraneous details (right) were both positively associated with reading comprehension difference scores (i.e., extent to which children’s reading comprehension improved in the Streamlined condition) suggesting that children who tended to look away from the text or tended to fixate on extraneous illustration details in the Standard condition, showed greater gains in comprehension when extraneous details were removed in the Streamlined condition. Shaded regions represent 95% confidence interval of the prediction line.

Next, we investigated whether reading in the Streamlined condition is especially useful for children with less developed attention regulation. For this analysis, a comprehension difference score for each child was calculated by subtracting the Standard condition comprehension score from the Streamlined condition score, such that higher and positive difference scores indexed greater gains in reading comprehension. Comprehension difference scores ranged from −57.14 to 85.71%, with a mean of 32.86% (SD = 31.38%). Higher gaze shifts away from the text *r*(57) = 0.63, 95% CI [0.45, 0.80], *p* < 0.0001 and higher fixations to extraneous illustrations, *r*(57) = 0.61, 95% CI [0.42, 0.75], *p* < 0.0005 were positively associated with how much children’s comprehension improved when reading in the Streamlined condition (see Fig. 5). Specifically, children who were more prone to look away from the text and who tended to fixate on extraneous illustrations while reading showed greater gains in comprehension when reading in the Streamlined condition.

To examine the extent to which fixations to extraneous details uniquely predicted how much children’s comprehension improved reading in the Streamlined condition, a multiple regression analysis was conducted that included extraneous illustration fixations, relevant illustration fixations, and WRI reading proficiency scores as predictors of children’s comprehension difference scores (see Table 2). The additive model accounted for 42.93% of the variability in comprehension difference scores (*F* = 13.79, *df* = 3, 55, *p* < 0.0001). The only significant predictor of comprehension difference scores was fixations to extraneous details (*β* = 0.95, *t* = 2.78, *p* = 0.007, 95% CI [0.27, 1.63]).

**Table 2:** Regression analysis predicting comprehension improvement from streamlined condition.

Item	*β*	SE	*t*	*p*	95% CI	*F*	df	*R*^2^
Model				<0.0001		13.79	3, 55	0.43
Extraneous fixations	0.95	0.34	3.78	0.007	[0.27, 1.63]			
Relevant fixations	0.38	0.49	0.79	0.435	[−0.59, 1.36]			
Reading proficiency (WRI)	−0.35	0.18	−1.91	0.061	[−0.71, 0.02]			

### Follow-up control experiment

We conducted a follow-up experiment with another sample of 1st and 2nd-grade students (*n* = 60) to examine the possibility that the results above may be due to making the text more discriminable against the background, rather than due to the removal of extraneous illustrations. Towards this goal, we created a new condition: a Featureless Background condition, in which the text was placed on a white background but no other changes were made to the illustrations (see Fig. 1). We then compared children’s gaze shifts and reading comprehension in the Standard condition to the Featureless Background condition, using the same within-subjects design and procedure as Experiment 1 (see “Methods”). The results of the control experiment revealed a main effect of grade on gaze shifts away from the text, *F*(1, 57) = 8.44; *p* = 0.005; Cohen’s *d* = 0.71. The fixed intercept value of 16.95 represents the mean gaze shifts away from the text while reading in the Featureless Background condition. The intercept for gaze shifts away from the text for first-grade children is 16.95 + 11.15 = 28.10, and this is significantly higher than the mean gaze shifts away from the text for second-grade children (*t* = 2.91, *p* = 0.005, 95% CI for the difference is 3.47–18.84 higher). These findings reproduce a similar trend from Experiment 1 of first-grade children looking away from the text more frequently than second-grade children. There was no main effect of book condition (Cohen’s *d* = 0.07) nor order (Cohen’s *d* = 0.03) on mean gaze shifts away from text and reading comprehension scores (all *F*s < 1.4, all *p*s > 0.24), ruling out the possibility that the observed effects in the Streamlined condition stemmed from enhancing text salience (see Table S7 and Table S8 in the online Supplementary Material for LMM analyses with and without the interaction terms). The control experiment replicated the following findings of the main experiment: lower reading comprehension scores were associated with higher tendency to look away from the text (*r*(58) = −0.51, 95% CI [−0.67, −0.30], *p* < 0.0005) and higher fixations to extraneous details (*r*(58) = −0.48, 95% CI [−0.65, −0.26], *p* < 0.0005). The results of the control experiment provide evidence that the effects of the Streamlined condition were not solely attributed to making the text more discriminable against the background. Results of the control experiment also replicate findings of the main experiment of the negative association between children’s eye-gaze patterns and reading comprehension. Full details of the implementation, method, and results from the control experiment are reported in the Supplementary Materials (pp. 10–19).

## Discussion

The reported results provide evidence that excluding extraneous illustrations from reading materials for beginning readers can enhance children’s attention to the text and improve reading comprehension. These findings are strengthened further by the results of the follow-up control experiment, which provided evidence that the benefits of removing extraneous illustrations for attention and reading comprehension were unlikely to be driven by greater text discriminability. Nearly all children exhibited fewer gaze shifts away from the text and obtained higher comprehension scores when reading in the Streamlined condition compared to the commercially available Standard condition. Furthermore, children who frequently shifted their gaze away from the text and fixated on extraneous details while reading in the Standard condition (presumably, due to less developed attentional control) exhibited the greatest gains in comprehension from reading in the Streamlined condition. Importantly, the regression model revealed that the associations between children’s eye-gaze patterns and comprehension were not entirely due to variance shared with overall reading proficiency or the ability to match words with referents: fixations to extraneous illustrations was the only significant predictor of gains in reading comprehension, while reading proficiency (WRI scores) and fixations to relevant illustrations were not.

In the Standard condition, children made frequent gaze shifts away from the text to the illustrations. Frequent switching between two different tasks—reading the text to understand the story on one hand and exploring engaging illustrations on the other hand—might place too much extraneous load on young children’s working memory resulting in decreased reading comprehension^46^. Because illustrations matched the story text in the Streamlined condition (i.e., illustrations reinforced the text without extraneous load), children did not have an opportunity to encode illustration details that were irrelevant to the text. Instead, the relevant illustrations may have helped children integrate nonverbal information and text to develop a better representation of the story (for relevant findings with proficient readers see refs. ^47,48^). Future research is needed to test this possibility by comparing beginning readers’ comprehension in a book that contains only relevant illustrations and no illustrations, a possibility we are currently exploring.

The inclusion of only relevant illustrations may be particularly beneficial for children who frequently look away from the text because these children’s ability to selectively attend to relevant information while suppressing irrelevant, extraneous information is less efficient. Prior findings suggest that children’s attentional control and ability to focus in preschool and first grade are significant predictors of reading achievement years later in fourth grade and even into adulthood at age 21^49,50^. These results point to the importance of taking attentional control—a foundational component linked to school readiness and reading achievement—into account when designing educational materials.

One limitation to this study is that the reading comprehension assessment primarily focused on the recall of key story events, and as such assessed both understanding and memory of the story. In future research, it would be important to incorporate multiple assessments of comprehension, including assessments that have lower memory demands. Another limitation is that this study did not include an independent measure of attention. Future studies should include independent measures of attention to examine whether modifying aspects of the book design is generally beneficial for beginning readers or whether this instructional support is particularly promising for specific populations of children.

In summary, the results of this study show that extraneous illustrations details increase gaze shifts away from text and decrease reading comprehension in beginning readers. Furthermore, higher fixations to extraneous illustrations during reading were associated with lower reading comprehension scores. It is important to note that we are not advocating for the removal of illustrations from books, but rather encouraging consideration of whether and how the design of instructional reading materials for beginning readers can be optimized by taking into account children’s developing attention regulation skills. In addition, the motivational and engaging aspects of illustrations in books for beginning readers should not be ignored: it is well-known that children like pictures. Alice, the beloved character of Charles Dodgson’s story Alice’s Adventures in Wonderland^51^, famously wondered “what is the use of a book … without pictures?” In the present study the effect of removing extraneous illustrations on children’s engagement was not measured due to the nature of the within-subject design. However, children’s total looking duration and looking duration to pictures in the Streamlined and Standard conditions were approximately equal. Nevertheless, it remains to be explored in future research, whether removing extraneous illustration details may affect children’s motivation.

The nature of illustrations that accompany text may have important implications for attention and learning not just for students who are reading-to-learn but also for children who are learning-to-read. The findings presented here highlight the opportunity to improve the design of educational materials for beginning readers by limiting extraneous illustrations in order to better support children’s developing attentional regulation and reading comprehension. These findings suggest that enhancements to instructional reading materials should serve a clear purpose to engage the child with the story content while ensuring they do not interfere with performance and learning. The consideration of the potential costs of adding extraneous illustrations may be especially important for children with less developed attention regulation.

## Methods

### Participants

Sixty-six participants were recruited; however, only children who exhibited a minimum level of decoding proficiency on an independent measure of reading fluency (i.e., passed Level 1 on the Word Recognition in Isolation measure described below) continued with the study. Children who did not show the minimum level of reading proficiency to continue in the study, read a simpler book with the experimenter. The final sample consisted of 60 children (*M*_age_ = 7.56 years, SD = 7 months; 27 females, 24 males, and 9 children whose sex was not reported) in Grade 1 (*n* = 30) and Grade 2 (*n* = 30). See Table S2 in the online Supplementary Material for mean age and sex of participants by grade level. Primary school children were targeted for the present study as young children were hypothesized to be particularly susceptible to attentional competition between text and engaging illustrations due to the combination of their immature attention regulation system and developing decoding skills. Participants were recruited from schools in and around a Mid-Atlantic city in the United States. The race and ethnicity information for the sample reported by the parents was as follows: 41.7% White, 40.0% African American or Black, 10.0% Multi-Racial, 1.7% reported as Other, and 6.7% unreported. The experimental protocol was approved by the Carnegie Mellon University Institutional Review Board (protocol STUDY2017_00000301). Signed consent was obtained from the parents of participants. Children were tested individually by hypothesis-blind trained research assistants and children were given a small prize for their participation (e.g., a bouncy ball or marble maze toy).

### Design, materials, and procedure

To maintain a high level of ecological validity, children read a commercially available book entitled *Good Job Dennis* from the “Hooked on Phonics®” curriculum for first grade (“Hooked on Phonics®” is a trademark of Sandviks HOP, Inc. This publication is not sponsored or endorsed by Sandviks HOP, Inc.). Detailed descriptions of the materials (including minor modifications to the book to equate the number of words across conditions), instructions, and procedure are provided in the Supplementary Materials. A brief overview is provided below. See Table S4 and Table S6 in the Online Supplementary Material for descriptive statistics on the reading and eye-tracking outcome measures by book condition.

### Preliminary study: the classification of extraneous illustration details

A calibration study with adults (*n* = 15) was conducted to determine which illustrations were relevant and which were extraneous. Participants were presented with the book in the Standard layout and were given instructions to outline the details in the illustrations they believed were relevant to the story. The illustration details that participants reached over 90% agreement on were considered relevant illustrations and retained in the Streamlined condition, whereas the other illustration details were deemed extraneous illustrations and excluded.

The book condition was manipulated within-subjects: half of the book was presented to children in a commercially available “Standard” condition, and in the other half of the book the extraneous illustrations were removed (“Streamlined” condition). Minor modifications were made to the text to ensure each half of the book matched in length (see the online Supplementary Material for detailed explanation). The final version of the book used in this study contained a total of 12 pages (six double-page spreads), resulting in 6 pages (three double-page spreads) per condition. The average number of words per double-page spread was 43.0 in the first half of the book and 42.3 in the second half of the book. The book was presented on a laptop computer. Reading was self-paced, and participants advanced to the next screen by pressing a button on the keyboard. After reading the story, children’s reading comprehension was assessed. Note that children were informed that they would be asked a few questions about the story. Each testing session was videotaped with a Logitech C920 HD Pro Webcam. Testing sessions mimicked a guided-reading instructional session that children typically encounter when reading with a teacher. Thus, the experimenter scaffolded children’s decoding. For instance, when children made a decoding error or skipped a word, the experimenter prompted the child to try again and when necessary helped the child sound out the word. All prompts were recorded. These scaffolds were implemented to align with current literacy instruction practices and served to minimize children’s frustration.

### Measures

#### Eye-tracking measures

**Fig. 6:**
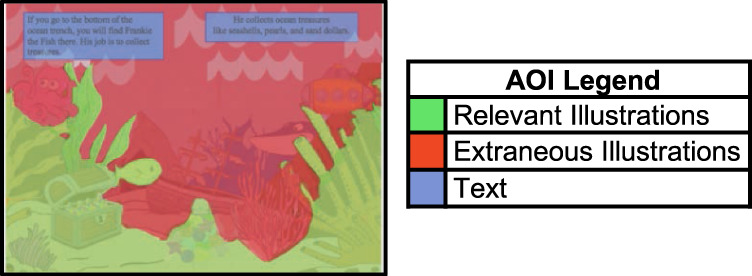
Areas of interest classification for eye gaze pattern analysis. Schematic depiction of the classification of areas of interest (AOIs) used for eye gaze pattern analysis. We used three AOI categories: Relevant Illustration AOIs (in green)—illustrations reinforcing the text; Extraneous Illustration AOIs (in red)—illustration details not pertinent to the text; and Text AOIs (in blue). Note that this is an original image hand-drawn and developed by the first author of the study to schematically depict the AOI classifications used in this study. Actual images of the reading materials used in the study are not reproduced here to avoid copyright infringement. Examples of the AOIs drawn on the materials used in the study can be viewed in the Open Science Framework repository for the study: https://osf.io/frgw8/?view_only=42259f9134024b54bd5adae2da7f9c2a.

An SMI RED250 mobile eye tracker^52^ was used to measure children’s eye movements while reading. On each page of the book, we created areas of interest (AOIs) for text, illustrations, and white space. Relevant illustration AOIs were designated as the illustration details retained for the Streamlined condition (mean area covered by relevant illustrations was *M* = 686,807 pixels), while extraneous illustration AOIs were designated as the details omitted in the Streamlined condition (mean area covered by extraneous illustrations was *M* = 622,332 pixels; a schematic depiction of this classification is shown in Fig. 6; see Table S1 in the online Supplementary Material for total pixels per page, by AOI). SMI BeGaze Eyetracking Analysis Software was then used to calculate average gaze shifts away from the text, average fixations to extraneous illustrations, and average fixations to relevant illustrations.

#### Word recognition in isolation test (WRI)

The WRI—adapted from Morris^45^—was administered to children prior to reading the story. The WRI measures children’s ability to recognize and decode individual words. The measure consists of a series of word lists that are graded in difficulty. Scores were calculated as the number of words read accurately in 90 s out of 100 total possible words. The WRI has been shown to be a strong predictor of contextual and oral reading levels^53,54^.

#### Reading accuracy

While children read the book aloud, the experimenter manually recorded the child’s decoding accuracy for each word in the story using a running record^44^. The experimenter also recorded any prompts that were administered. For each condition, decoding accuracy was calculated as the percentage of correct responses.

#### Reading comprehension

Reading comprehension is a complex process that has been notoriously challenging to assess^55,56^. Asking open-ended recall questions about a story (e.g., asking individuals to recall the characters, settings, character goals and solutions from the narrative) is one of the most common approaches to reading comprehension assessments with young children^57–59^. Furthermore, early childhood educators also use open-ended recall questions as the primary instructional strategy for reading comprehension in school settings^60^. One limitation of this assessment and similar measures is that they assess both children’s understanding of the events described in the text and their memory of the events; nevertheless, open-ended recall questions are considered one of the most appropriate assessments of reading comprehension with elementary school children^57^. Following this common practice, we chose to assess children’s reading comprehension using the open-ended questions provided by the book publisher labeled for educators, parents, and children as “reading comprehension questions.” Although these questions probe both children’s understanding and memory of the story, for brevity and following the convention in the literature, we refer to this outcome measure as a ‘reading comprehension’ assessment in this manuscript.

The commercially available book used in this study incorporated six suggested questions to assess children’s reading comprehension. To preserve ecological validity, we used the comprehension questions suggested by the publisher with minor modifications that ensured the questions were linked to the content presented on specific pages, making it possible to clearly distinguish events from the first or second half of the book (see the online Supplementary Material for the comprehension assessment modifications). There were three questions for each half of the book (two 2-point questions, and one 3-point question). A total of 14 points were possible, 7 points per condition. For example, in the first half of the book the job of the main character, Dennis, is described; these story details are not part of the content in the second half of the book. For the 2-point story question, children were asked, “What is Dennis’ job?” Children received full credit if they identified that Dennis directs traffic and helps children cross the street, 1 point for a partial answer (e.g., he helps children), and 0 points if they failed to recall Dennis’ job or provided an incorrect response. In the second half of the book, various animals escape from a pet shop including cats, dogs, birds, rabbits, and frogs; these story details are not part of the content in the first half of the book. For the 3-point question, children were asked, “What animals get out of the pet shop?” Children received full credit if they correctly identified all of the animals that escaped, 2 points if they identified at least 3 animals, 1 point if they identified only 2 animals, and 0 points if they failed to recall the animals that escaped or provided an incorrect response. Reading comprehension was measured as the percentage of correct responses (out of 7 possible points in each condition). The story questions were scored twice by hypothesis-blind research assistants who were also blind to the participants’ condition assignment. Inter-rater reliability using Cohen’s kappa^61^ was 0.85, indicating substantial coder consistency.

Children were also asked to orally recount the story as an additional measure of reading comprehension. The retelling measure was administered before the comprehension questions. Overall, children struggled with retelling the story, consistent with findings reported in prior literature suggesting that even on-grade readers tend to retell few main ideas and text details without question prompts^62^. Due to concerns about the overall low level of performance on the retelling measure, we report the details on the retelling measure administration, scoring, and results in the Supplementary Materials (pp. 20–22).

### Statistical analyses

A LMM was applied with maximum likelihood method to determine the main effects of condition (Standard or Streamlined), grade, and condition order on gaze shifts away from the text and comprehension scores. A random intercept model was applied. The effect of condition was treated as a repeated measure with “Unstructured” as the repeated covariance type. Neither the condition × grade interaction nor the condition × order interaction was found to be significant during the model selection process. Therefore, no interaction terms were included in the final LMM analyses^63^. Follow-up pairwise comparison with Bonferroni confidence interval adjustment was used to compare mean gaze shifts away from the text and comprehension scores between conditions. Differences of means and 95% CIs were determined using the LMMs. Effect sizes were determined using Cohen’s *d* and calculated using mean differences from the mixed model, the standard deviation of the means, and the correlation between the two conditions for the within-subjects variables:

1 $$d = \frac{{M_1 - M_2}}{{\sqrt {\mathrm{SD}_1 + {\mathrm{SD}}_2 - \left( {2r\mathrm{SD}_1\mathrm{SD}_2} \right)} }}$$

and calculated using mean differences from the mixed model, the standard deviation of the means, and the sample sizes for the between-subjects variables^64^:

2 $$d = \frac{{M_1 - M_2}}{{\sqrt {\left( {n_1 - 1} \right){\mathrm{SD}_1^2} + \left( {n_2 - 1} \right){\mathrm{SD}_2^2}/n_1 + n_2 - 2} }}$$

Based on the Fisher r-to-z transformation, 95% CIs for Pearson Correlation Coefficients were calculated utilizing the Pearson product-moment correlation coefficient observed within the sample and the number of paired observations of the sample^65^. Alpha was set at 0.05 for all statistical tests. All tests for these and other analyses were two-tailed. All statistical analyses were conducted in IBM SPSS Statistics V26.

### Reporting summary

Further information on experimental design is available in the Nature Research Reporting Summary linked to this article.

## Supplementary information

Supplementary Material

Reporting Summary

## Data Availability

The data reported and sample videos of children’s eye gaze patterns during reading are accessible in the Open Science Framework repository^66^, 10.17605/OSF.IO/FRGW8, https://osf.io/frgw8/?view_only=42259f9134024b54bd5adae2da7f9c2a.

## Code availability

We used SPSS (v.26) to perform the analyses. The scripts used to analyze these data can be found in the Open Science Framework repository^66^, https://osf.io/frgw8/?view_only=42259f9134024b54bd5adae2da7f9c2a.
