# Supplementary material for: Keep it simple: streamlining book illustrations improves attention and comprehension in beginning readers
Source: NPJ Sci Learn. 2020 Sep 28;5:14. doi: 10.1038/s41539-020-00073-5 (PMC7522290; doi:10.1038/s41539-020-00073-5)
Supplement: Supplementary file 1 — Supplementary Material [file 41539_2020_73_MOESM1_ESM.pdf]

Supplementary Materials to manuscript *Keep It Simple: Streamlining Book Illustrations Improves Attention and Comprehension in Beginning Readers*

The supplementary materials serve to share details about the study protocol, materials, and additional analyses.

I. **Study Materials**

Book Modifications p. 2
Reading Comprehension Questions Modifications p. 3
Retelling Assessment p. 20

II. **Tables of Descriptive Statistics**

Table S1: AOI Sizes by Page p. 4
Table S2 Experiment 1 Demographic Information p. 5
Table S3 Control Experiment Demographic Information p. 6
Table S4 Experiment 1 Reading and Eye-tracking Measures p. 7
Table S5 Experiment 1 LMM with Interaction Terms p. 8
Table S6 Control Experiment Reading and Eye-tracking Measures p. 9
Table S7 Control Experiment LMM p. 14
Table S8 Control Experiment LMM with Interaction Terms p. 15

III. **Additional Analyses and Findings**

Control Experiment Methods and Results p. 10-19
Retelling Assessment Scoring and Results p. 20-22

IV. **References** p. 23

Book Modifications:

- The original book had 15 single pages (excluding the title page).
- We cut three pages to reduce the duration of the task and to have an even amount of pages per condition (6 pages per condition, 12 pages total)
- Pages 10, 11, and 13 were removed, as the removal of these pages had no effect on the plotline and the supplied comprehension questions did not inquire about any information contained on these pages.
- Seven out of the 12 pages remained identical to the original book. Minor modifications were made on 5 out of the 12 pages to ensure that each half of the book approximately matched in length and words per page. For example, on page 3, the original sentence read *"He helps kids cross the street. Dennis says "Stop! and traffic stops. Dennis says "Go!" and he steps out to let traffic pass."* This sentence was modified as follows for the present study: "*He helps kids cross the street. Dennis says "Stop!" and traffic stops*."

[Full transcriptions of book not shared publicly to avoid violation of the copyright holder's right of public distribution]

Reading Comprehension Questions Modifications

Book publisher provided six comprehension questions; we used these questions to assess reading comprehension in the present study. One publisher provided question was replaced based on findings from pilot testing. Questions were pilot tested with 1st and 2nd grade children to ensure questions were equally challenging between the two halves of the book (Questions 1-3 linked to pages 1-3 versus Questions 4-6 linked pages 4-6), with neither question set presenting floor or ceiling effects. After pilot testing, it was evident that children were giving identical responses for two of the questions suggested by the publisher. Therefore, the question linked to text on page 3 was replaced with a new question developed and pilot tested by the researchers. The text of the full comprehension assessment is not reproduced here to avoid violation of the copyright holder's right of public distribution.

Table S1 AOI Size by Page

	Area of Interest (AOI)			
	Extraneous	Relevant	Text	**Total Pixels/Page**
Page1	718969	636503	164085	1519557
Page 2	648977	710939	150751	1510667
Page 3	718547	634720	137245	1490512
Page 4	719315	662707	122416	1504438
Page 5	565775	514823	248180	1328778
Page 6	749260	574299	159543	1483102
**Mean [px]**	686807	622332	163703	1472842
**Total [px]**	**4120843**	**3733991**	**982220**	**8837054**

Table S2 Experiment 1 Demographic Information

Sample Size and Mean Age of Participants in Years by Grade Level and Sex

	All		Female		Male		Unreported
Grade	*n*	*M (SD)*	*n*	*M (SD)*	*n*	*M (SD)*	*n*
1	30	7.10 (*.32*)	16	7.18 (*.28*)	12	7.01 (*.37*)	2
2	30	8.03 (*.39*)	11	7.94 (*.41*)	12	7.99 (*.37*)	7

Table S3 Control Experiment Demographic Information

Sample Size and Mean Age of Participants in Years by Grade Level and Sex

	All		Female		Male		Unreported
Grade	*n*	*M (SD)*	*n*	*M (SD)*	*n*	*M (SD)*	*n*
1	29	6.82 (*.38*)	13	6.70 (*.31*)	15	6.94 (*.42*)	1
2	31	7.94 (*.34*)	18	7.96 (*.32*)	9	7.91 (*.25*)	4

Note: There was one reporting error of the birthdate of one first grade participant (parent marked birthdate as the current year instead of birth year).

Table S4 Experiment 1

Mean (*SD*) of *Reading and Eye-tracking Measures, by Book Condition*

Measure *M* (*SD*)	Standard Condition	Streamlined Condition
Running Record (%)	95.79 (4.13)	95.84 (4.24)
Comprehension Raw Scores	3.32 (1.78)	5.62 (1.31)
Comprehension (% of 7)	47.38 (25.43)	80.24 (18.80)
Retell Raw Scores	2.03 (1.13)	2.43 (1.13)
Retell (% of 5)	40.67 (22.69)	48.68 (22.51)
Gaze Shifts Away From Text/Page	23.02 (20.77)	10.72 (9.29)
Relevant Illustration Fixations	12.72 (10.96)	13.84 (10.01)
Extraneous Illustration Fixations	16.27 (15.35)	/
Illustration Duration/Page (ms)	10178 (9852)	9371 (6089)
Relevant Illustration Duration/Page (ms)	4078 (4026)	9371 (6089)
Extraneous Illustration Duration/Page (ms)	5589 (5990)	/
Text Duration/Page (ms)	31511 (26369)	30314 (29418)
Reading Time/Page (ms)	42339 (30459)	40326 (31856)

Note: Looking Time at Illustrations/Page and Relevant Illustrations are the same in the Streamlined Condition because there are no Extraneous Illustrations

Table S5

Experiment 1 Estimates of Fixed Effects Obtained using the LMM with Interaction Terms

Outcome Variable	Parameter	Estimate	*SE*	*df*	*t*	*p*	95% CI
Gaze Shifts Away From Text	Intercept	4.57	3.59	85.34	1.27	.207	[-2.57, 11.71]
	[Condition=Standard][a]	12.26	3.37	56	3.63	.0006	[5.59, 19.02]
	[Grade=1][b]	4.37	4.08	85.34	1.07	.287	[-3.74, 12.47]
	[Order=Standard First][c]	7.48	4.08	85.34	1.83	.070	[-6.34, 15.60]
	[Condition x Grade][d]	5.27	3.83	56	1.38	.174	[-2.40, 12.94]
	[Condition x Order][e]	-5.01	3.83	56	-1.31	.197	[-12.69, 2.67]
Comprehension Scores (%)	Intercept	78.10	5.04	114	18.59	<.0005	[68.12, 88.09]
	[Condition=Standard][a]	-29.69	7.12	57	-4.17	.0001	[-43.95, -15.44]
	[Grade=1][b]	-2.60	4.85	114	-0.54	.656	[-14.14, 8.93]
	[Order=Standard First][c]	6.65	4.85	114	1.37	.256	[-4.89, 18.19]
	[Condition x Grade][d]	-1.76	8.22	57	-0.21	.832	[-18.22, 14.71]
	[Condition x Order][e]	-4.42	8.23	57	-0.54	.593	[-20.90, 12.05]

[a]The LMM model used results from Standard Condition as the reference.
[b]The LMM model used results from Grade 1 as the reference.
[c]The LMM model used results from the Order of the Standard Condition presented first as the reference.
[d]The LMM model used results from Grade 1 as the reference and the Standard Condition as the references.
[e]The LMM model used results from the Order of the Standard Condition presented first and the Standard Condition as the references.

Table S6 Control Experiment

Mean (*SD*) of *Reading and Eye-tracking Measures, by Book Condition*

Measure	Standard	Featureless Background
Running Record (%)	95.57 (4.27)	96.57 (4.22)
Comprehension Raw Scores	4.58 (1.72)	4.72 (1.73)
Comprehension (% of 7)	65.48 (24.57)	67.38 (24.57)
Retell Raw Score	2.15 (1.05)	2.17 (1.14)
Retell (% of 5)	43.00 (21.10)	43.33 (22.75)
Gaze Shifts Away From Text/Page	21.04 (17.39)	21.90 (15.92)
Relevant Illustration Fixations	12.41 (11.30)	12.31 (10.80)
Extraneous Illustration Fixations	16.10 (14.90)	17.05 (15.30)
Illustration Duration/Page (ms)	10441 (9688)	11235 (10721)
Relevant Illustration Duration/Page (ms)	3873 (3914)	3802 (3711)
Extraneous Illustration Duration/Page (ms)	5542 (5794)	6262 (7267)
Text Duration/Page (ms)	28239 (26224)	30972 (25126)
Reading Time/Page (ms)	39332 (29572)	42674 (28639)

Control Experiment

This study tested the alternative hypothesis that the results observed in the main experiment may be due, in whole or in part, to manipulating the figure-ground text discrimination, rather than reducing attention to extraneous illustrations. In this control experiment, we used the same book as in the main experiment and presented it to children in the commercially available Standard condition. However, in the control experiment, we added a new condition in which we increased text discriminability by placing it on a featureless background (Featureless Background condition). If the effects on comprehension observed in Experiment 1 were due to increased text discriminability, then we should observe higher reading comprehension scores in the Featureless Background condition than in the Standard condition. However, if the effects on reading comprehension observed in Experiment 1 were due primarily to the removal of extraneous illustrations, then we should not observe improved reading comprehension in the Featureless Background condition compared to the Standard condition.

Method

Participants

Seventy-six participants were recruited (none of whom participated in Experiment 1) for Experiment 2. As in Experiment 1, only children who passed Level 1 on the WRI continued with the study. A total of 16 children did not meet the minimum proficiency level and thus were not included in the present study (as in Experiment 1, these children read a simpler book with the experimenter). The final sample consisted of 60 children (M_{age}= 7.39 years, SD = 8 months, 31 females, 24 males, and 5 children whose sex was not reported) in Grade 1 (n = 29) and Grade 2 (n = 32). See Table S3 for mean age and sex of participants by grade level. Participants were recruited from schools in and around a Mid-Atlantic city in the United States. Information on the

children's race and ethnicity was provided by their parents as follows: 63.3% of the children were White, 8.3% African American or Black, 15.0% Multi-Racial, 1.7% East Asian or Asian American, 1.7% South Asian or Indian American, and 10% were not reported. Signed consent was obtained from the participants' parents. Children were tested individually by hypothesis-blind trained research assistants and children were given a small prize for their participation.

Design and Materials

The procedure and apparatus (eye-tracking equipment, reader protocol) were identical to those described in Experiment 1. The materials and design were nearly identical to those of Experiment 1. The book condition was again manipulated within-subjects: half of the book was presented to children in a commercially available "Standard" condition; however, in the control experiment, a new experimental condition was used in which the text was presented on a featureless background. The order of the conditions (Standard first vs. Featureless Background first) was randomly assigned and counterbalanced across participants.

Measures

Eye-tracking measures, reading accuracy, reading proficiency, and reading comprehension (Cohen's Kappa = .91)[1] were identical to the measures described in Experiment 1. As in Experiment 1, the story retelling measure was administered; details on the administration, scoring, and results on this measure are reported below.

Results

Reading Level

Children were beginning readers as evidenced by their performance on the WRI, the independent measure of children's reading proficiency (M = 67.18; SD = 18.31). Similar to Experiment 1, the difficulty level of the book was considered appropriate for the participants as evidence by the

high mean Running Record scores (*M* = 96.56%; *SD* = 4.30%). As in Experiment 1, the manipulation to the book condition did not influence children's decoding accuracy (Standard: *M* = 96.57%; *SD* = 4.27%; Featureless Background: *M* = 96.55%; *SD* = 4.32%), paired-sample $t(59) = 1.45$, $p = .15$, Cohen's $d = 0.19$.

Eye-tracking Results

There was no significant difference in the total looking duration in the Standard condition (*M* = 39,332 ms; *SD* = 29,572 ms) compared to the Featureless Background condition (*M* = 42,674 ms; *SD* = 28,639 ms), paired-sample $t(59) = 1.97$, $p = .053$, Cohen's $d = 0.25$.

Gaze Shifts To assess possible order effects and grade differences, we conducted a linear mixed-effects model (LMM) on gaze shifts away from the text, with book condition, grade, and order modeled as fixed effects and subject as a random effect. Table S7 shows the estimations of fixed effects and the corresponding 95% confidence intervals (CI). There was a main effect of grade on gaze shifts away from text, $F(1, 57) = 8.44$; $p = .005$; Cohen's $d = .71$. The fixed intercept value of 16.95 represents the mean gaze shifts away from the text while reading in the Featureless Background condition. The intercept for gaze shifts away from the text for first grade children is 16.95 + 11.15 = 28.10, and this is significantly higher than the mean gaze shifts away from the text for second grade children ($t = 2.91$, $p = .005$, 95% CI for the difference is 3.47 to 18.84 higher). There was no main effect of book condition, $F(1, 59) = .33$, $p = .568$; Cohen's $d = .07$, or order, $F(1, 57) = .05$, $p = .82$; Cohen's $d = .03$, and no significant interactions between any of these factors and gaze shifts away from the text (all *p*s > .10; see Table S8 for the LMM analysis with the interaction terms).

Reading Comprehension To assess possible order effects and grade differences, we conducted a LMM on reading comprehension, with book condition, grade, and order modeled as fixed

effects and subject as a random effect. Table S7 shows the estimations of fixed effects and the corresponding 95% CIs. There was no main effect of book condition, $F(1, 59) = .40$, $p = .532$; Cohen's $d = .08$, order, $F(1, 57) = .14$, $p = .707$; Cohen's $d = .07$, or grade, $F(1, 57) = 1.38$, $p = .245$; Cohen's $d = .26$, and no significant interactions between any of these factors and reading comprehension (all ps > .08; see Table S8 for the LMM analysis with the interaction terms). These results indicate that children's mean comprehension scores when reading in the Featureless Background condition did not differ from their comprehension scores in the Standard condition, regardless of grade or order in which the book conditions were presented. Therefore, we found no support for the possibility that differences observed in Experiment 1 stemmed from increasing text discriminability rather than from removing extraneous illustrations.

Table S7

Control Experiment Estimates of Fixed Effects Obtained using the LMM

Outcome Variable	Parameter	Estimate	*SE*	*df*	*t*	*p*	95% CI
Gaze Shifts Away From Text	Intercept	16.95	3.33	62.99	5.09	<.0005	[10.29, 23.61]
	[Condition=Standard][a]	-.855	1.49	59.00	-.574	.568	[-3.84, 2.13]
	[Grade=1][b]	11.15	3.84	57.00	2.91	.005	[3.47, 18.84]
	[Order=Standard First][c]	-.883	3.84	57.00	-.230	.819	[-8.56, 6.80]
Comprehension Scores (%)	Intercept	69.51	4.99	68.43	13.92	<.0005	[59.54, 79.48]
	[Condition=Standard][a]	-1.90	3.03	59.00	-.629	.532	[-7.96, 4.15]
	[Grade=1][b]	-6.60	5.62	57.00	-1.17	.245	[-17.86, 4.66]
	[Order=Standard First][c]	2.12	5.62	57.00	.378	.707	[-9.13, 13.38]

[a]The LMM model used results from Standard Condition as the reference.
[b]The LMM model used results from Grade 1 as the reference.
[c]The LMM model used results from the Order of the Standard Condition presented first as the reference.

Table S8

Control Experiment Estimates of Fixed Effects Obtained using the LMM with Interaction Terms

Outcome Variable	Parameter	Estimate	*SE*	*df*	*t*	*p*	95% CI
Gaze Shifts Away From Text	Intercept	17.76	3.48	73.58	5.10	<.0005	[10.82, 24.70]
	[Condition=Standard][a]	-2.48	2.51	57	-0.99	.327	[-7.50, 2.54]
	[Grade=1][b]	12.01	4.11	73.58	2.92	.005	[3.81, 20.20]
	[Order=Standard First][c]	-3.33	4.11	73.58	-0.81	.420	[-11.53, 4.86]
	[Condition x Grade][d]	-1.71	2.96	57	-0.58	.565	[-7.64, 4.21]
	[Condition x Order][e]	4.90	2.96	57	1.66	.103	[-1.02, 10.82]
Comprehension Scores (%)	Intercept	67.83	5.39	86.88	12.58	<.0005	[57.12, 78.55]
	[Condition=Standard][a]	1.45	5.07	57	0.29	.776	[-8.70, 11.59]
	[Grade=1][b]	-8.62	6.37	86.88	-1.35	.179	[-21.28, 4.04]
	[Order=Standard First][c]	7.43	6.37	86.88	1.17	.246	[-5.22, 20.08]
	[Condition x Grade][d]	4.04	5.98	57	0.68	.502	[-7.94, 16.02]
	[Condition x Order][e]	-10.61	5.98	57	-1.77	.081	[-22.59, 1.36]

[a]The LMM model used results from Standard Condition as the reference.
[b]The LMM model used results from Grade 1 as the reference.
[c]The LMM model used results from the Order of the Standard Condition presented first as the reference.
[d]The LMM model used results from Grade 1 as the reference and the Standard Condition as the references.
[e]The LMM model used results from the Order of the Standard Condition presented first and the Standard Condition as the references.

Association between Eye Gaze Patterns and Reading Comprehension

We next examined the association between reading comprehension scores with gaze shifts away from the text and fixations to extraneous details, to assess the replicability of the findings reported in Experiment 1. For this analysis, total comprehension scores for each child were calculated by collapsing the Standard comprehension score and the Featureless Background comprehension score because there was no main effect of book condition on comprehension (see Fig. S2 and Fig. S3 for the association between comprehension scores and attention allocation while reading, by condition). Thus, total comprehension scores were calculated as the percentage of correct responses out of 14 possible points. Total comprehension scores ranged from 14.29% to 100.00%, with a mean of 66.43% (*SD* = 21.66%). Similarly, we collapsed average fixations to extraneous illustrations across the Featureless Background and Standard conditions (*M* = 16.56; *SD* = 14.48). Similar to Experiment 1, increased gaze shifts away from the text, $r(58) = -0.51$, 95% CI [-.67, -.30], $p < .0005$, and higher fixations to extraneous details $r(58) = -.48$, 95% CI [-.65, -.26], $p < .0005$, were negatively associated with children's comprehension scores (see Fig. S1). In other words, children who were more likely to look away from the text and fixate on extraneous details while reading also tended to have lower reading comprehension scores. These findings replicate the results from Experiment 1 in that children who are prone to attend to extraneous illustrations while reading have lower reading comprehension scores.

Fig. S1

Scatterplots for Association between Reading Comprehension and Eye Gaze Patterns in the Control Experiment

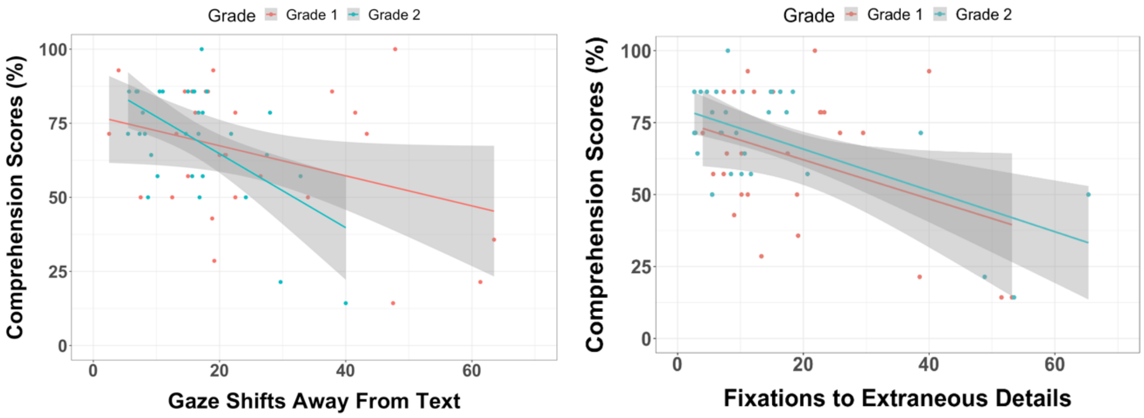

a b

Scatterplots of correlations between reading comprehension scores (% correct) and eye gaze patterns collapsed across conditions. Higher gaze shifts away from the text (left) and higher fixations to extraneous details (right) were both negatively associated with reading comprehension scores. Shaded regions represent 95% confidence interval of the prediction line.

Control Experiment Comprehension Scores and Eye Gaze Patterns, by Condition

Fig. S2

Scatterplots for Association between Reading Comprehension and Eye Gaze Patterns in the Control Experiment Standard Condition

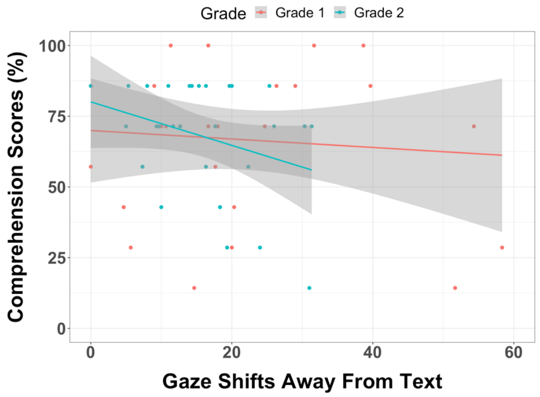

a

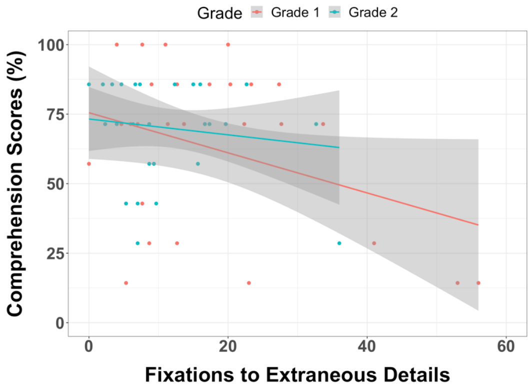

b

Scatterplots of correlations in the Control Experiment Standard Condition between reading comprehension scores (% correct) and gaze shifts away from the text (on left) and fixations to extraneous details (on right). Higher gaze shifts away from the text, $r(58) = -0.38$, $p = .003$, and higher fixations to extraneous details $r(58) = -.38$, $p = .003$, were negatively associated with children's comprehension scores in the Standard condition. Shaded regions represent 95% confidence interval of the prediction line.

Fig. S3

Scatterplots for Association between Reading Comprehension and Eye Gaze Patterns in the Control Experiment Featureless Background Condition

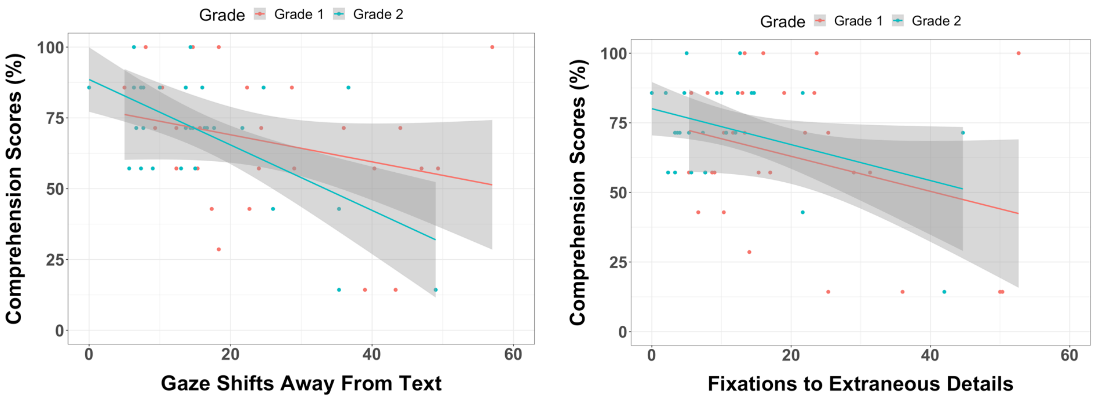

a b

Scatterplots of correlations in the Control Experiment Featureless Background condition between comprehension scores (% correct) and gaze shifts away from the text (left) and fixations to extraneous details (right). Higher gaze shifts away from the text, $r(58) = -0.54$, $p < .0005$, and higher fixations to extraneous details, $r(58) = -.46$, $p < .0005$, were negatively associated with children's comprehension scores in the Featureless Background condition. Shaded regions represent 95% confidence interval of the prediction line.

Retelling Assessment

Child ID: ___________________ DOB: _________________ DOT: _____________

Instructions: Ask the child to tell you what happened in the story. If the child needs help getting started, it is ok to ask them what happened first (or to use similar prompts: "then what happened?"/ "What happened next?" / "What happened last?"). Record the child's response. If you provide any prompts for clarification or prompts for additional information, please note that as well on the score sheet.

Child's Retelling:

Retelling Key Events: Retelling is a common measure of reading comprehension in elementary school, although recall accuracy for main ideas and details is often low even for on-grade readers (for review see see ref. [2]). Children were asked to orally recount the story and their response was transcribed verbatim by the experimenter. There were a total of 10 possible events to recount, five events per condition. Retelling was scored twice by hypothesis-blind research assistants who were also blind to the participants' condition assignment. Inter-rater reliability was .92 in Experiment 1 and .94 in the Control Experiment, indicating substantial coder consistency. Story retelling was measured as the percentage of correct events recounted (out of 5 possible events in each condition).

Experiment 1: Retelling Results

To assess possible order effects and grade differences, we conducted a LMM on retelling scores, with book condition, grade, and order modeled as fixed effects and subject as a random effect. There was a main effect of book condition, $F(1, 59) = 5.04$; $p = .029$; Cohen's $d = .29$. The fixed intercept value of 52.99 represents the mean retelling scores (in %) for the Streamlined Condition. The intercept for retelling scores in the Standard Condition is 52.99 – 8.00 = 44.99, and this is significantly lower than retelling scores in the Streamlined Condition ($t = -2.25$, $p = .029$, 95% CI for the difference is 0.87% to 15.13% lower). Follow-up pairwise comparisons after Bonferroni corrections revealed that on average, children scored 8.00% ($SE = 3.56$) higher on the retelling assessment in the Streamlined condition compared to the Standard condition. There was a main effect of grade, $F(1, 57) = 4.25$, $p = .044$; Cohen's $d = .42$. The intercept for retelling scores for first grade children is 52.99 – 9.36 = 43.63, and this is significantly lower than mean retelling scores for second grade children ($t = 2.06$, $p = .044$, 95% CI for the difference is 0.27% to 18.44% lower). There was no main effect of order, $F(1, 57) = .02$, $p =$

.884; Cohen's d = .02. These findings support the prediction that retelling scores would be higher in the Streamlined condition than in the Standard condition.

Control Experiment: Retelling Results

To assess possible order effects and grade differences, we conducted a LMM on retelling scores with book condition, grade, and order modeled as fixed effects and subject as a random effect. There was a main effect of grade on retelling scores, $F(1, 57) = 6.10$; $p = .017$; Cohen's $d = .54$. The fixed intercept value of 47.24 represents the mean retelling scores (in %) in the Featureless Background condition. The intercept for retelling scores for first grade children is 47.24 – 11.58 = 35.66, and this is significantly lower than the mean retelling scores for second grade children ($t = -2.47$, $p = .017$, 95% CI for the difference is 2.19% to 20.97% lower). There was no main effect of book condition, $F(1, 59) = .01$, $p = .909$; Cohen's $d = .02$, or order, $F(1, 57) = .52$, $p = .473$; Cohen's $d = .13$.

References

1. Cohen, J. A coefficient of agreement for nominal scales. *Educational and Psychological Measurement*. 20, 37–46, http://dx.doi.org/10.1177/001316446002000104, (1960).
2. Nilsson, N. L. A Critical Analysis of Eight Informal Reading Inventories. *The Reading Teacher*. 61, 7, 526–536, https://doi.org/10.1598/rt.61.7.2, (2008).
