# Supplementary material for: Keep it simple: streamlining book illustrations improves attention and comprehension in beginning readers
Source: NPJ Sci Learn. 2020 Sep 28;5:14. doi: 10.1038/s41539-020-00073-5 (PMC7522290; doi:10.1038/s41539-020-00073-5)
Supplement: Supplementary file 2 — Reporting Summary [file 41539_2020_73_MOESM2_ESM.pdf]

nature research

Corresponding author(s): Cassondra Eng

Last updated by author(s): Jun 8, 2020

Reporting Summary

Nature Research wishes to improve the reproducibility of the work that we publish. This form provides structure for consistency and transparency in reporting. For further information on Nature Research policies, see our Editorial Policies and the Editorial Policy Checklist.

Statistics

For all statistical analyses, confirm that the following items are present in the figure legend, table legend, main text, or Methods section.

n/a	Confirmed	
☐	☒	The exact sample size (n) for each experimental group/condition, given as a discrete number and unit of measurement
☐	☒	A statement on whether measurements were taken from distinct samples or whether the same sample was measured repeatedly
☐	☒	The statistical test(s) used AND whether they are one- or two-sided<br>*Only common tests should be described solely by name; describe more complex techniques in the Methods section.*
☐	☒	A description of all covariates tested
☐	☒	A description of any assumptions or corrections, such as tests of normality and adjustment for multiple comparisons
☐	☒	A full description of the statistical parameters including central tendency (e.g. means) or other basic estimates (e.g. regression coefficient) AND variation (e.g. standard deviation) or associated estimates of uncertainty (e.g. confidence intervals)
☐	☒	For null hypothesis testing, the test statistic (e.g. F, t, r) with confidence intervals, effect sizes, degrees of freedom and P value noted<br>*Give P values as exact values whenever suitable.*
☒	☐	For Bayesian analysis, information on the choice of priors and Markov chain Monte Carlo settings
☒	☐	For hierarchical and complex designs, identification of the appropriate level for tests and full reporting of outcomes
☐	☒	Estimates of effect sizes (e.g. Cohen's d, Pearson's r), indicating how they were calculated

Our web collection on statistics for biologists contains articles on many of the points above.

Software and code

Policy information about availability of computer code

Data collection	SensoMotoric Instruments (SMI) BeGaze software was used to acquire eye tracking data.
Data analysis	IBM SPSS Statistics V26

For manuscripts utilizing custom algorithms or software that are central to the research but not yet described in published literature, software must be made available to editors and reviewers. We strongly encourage code deposition in a community repository (e.g. GitHub). See the Nature Research guidelines for submitting code & software for further information.

Data

Policy information about availability of data

All manuscripts must include a data availability statement. This statement should provide the following information, where applicable:

- Accession codes, unique identifiers, or web links for publicly available datasets
- A list of figures that have associated raw data
- A description of any restrictions on data availability

The data reported in this manuscript and sample videos of children's eye gaze patterns during reading are available in the Open Science Framework repository, https://osf.io/frgw8/?view_only=42259f9134024b54bd5adae2da7f9c2a.

Field-specific reporting

Please select the one below that is the best fit for your research. If you are not sure, read the appropriate sections before making your selection.

☐ Life sciences ☒ Behavioural & social sciences ☐ Ecological, evolutionary & environmental sciences

For a reference copy of the document with all sections, see nature.com/documents/nr-reporting-summary-flat.pdf

Behavioural & social sciences study design

All studies must disclose on these points even when the disclosure is negative.

Study description	This is a quantitative behavioral study using a mixed experimental design, with Experimental Condition as a within-subject variable and Grade as a between-subject variable.
Research sample	The sample included 1st and 2nd Grade children from the Pittsburgh, PA area. The sample was not representative. Every effort was made to recruit a diverse sample of participants in terms of gender, race, and Socio-Economic Status. In Experiment 1, participants were 60 children (Grade 1, n = 30; Grade 2, n = 30; 27 females, 24 males, and 9 children whose sex was not reported; 41.7% of the participants were White, 40.0% African American or Black, 10.0% Multi-Racial, 1.7% reported their race as Other, and 6.7% were not reported). In Experiment 2, participants were 60 children (Grade 1, n = 29; Grade 2, n = 31; 31 females, 24 males, and 5 children whose sex was not reported; 63.3% of the participants were White, 8.3% African American or Black, 15.0% Multi-Racial, 1.7% East Asian or Asian American, 1.7% South Asian or Indian American, and 10% were not reported).
Sampling strategy	Convenience sample was recruited for the study. We contacted several schools in the Pittsburgh area, and schools that agreed to partner with us advertised the study to parents. Children's parents or legal guardians then volunteered to have their child participate in the study. Every effort was made to recruit children from a diverse set of schools, including public charter schools and private schools in different neighborhoods across Pittsburgh. We deemed 60 children per study to be sufficient sample size on the basis of pilot work.
Data collection	Trained research assistants blind to the hypotheses collected the data. Blinding to the Experimental condition was not possible due to the nature of the study (i.e., researchers read a book with the child, therefore they could see whether the child was looking at pages in the Standard or Streamlined condition). A laptop computer outfitted with a built-in webcam and a mobile SMI RED 250 eye tracker, and paper & pen were used to collect data. A laptop was used to present book pages to children; a built-in webcam was used to record video of the testing session; a mobile eye tracker was used to record children's gaze patterns; paper & pencil were used to record children's answers to Comprehension questions. The story questions were scored twice by hypothesis-blind research assistants who were also blind to the participants' condition assignment. Researchers tested children in a quiet place at their school; no one else was present during the testing session.
Timing	Data collection started on 04/25/2017 and ended 12/12/2018.
Data exclusions	In Experiment 1, eye-tracking data from 1 participant were not included in the analyses due to a technical failure. Participants were excluded from the study based on a predetermined criterion: only children who exhibited a minimum level of decoding proficiency on an independent measure of reading fluency (i.e., passed Level 1 on the Word Recognition in Isolation measure) were included in the study. In Experiment 1, 6 out of 66 children were excluded from the study, and in Experiment 2, 16 out of 76 children did not meet the minimum proficiency level and thus were excluded from the study.
Non-participation	No participants declined participation in either Experiment 1 or Experiment 2.
Randomization	For the between-subject variable Grade, no randomization was possible. The Experimental condition variable was manipulated within-subjects, such that each child participated in both conditions with the order of conditions counterbalanced across participants and randomly assigned.

Reporting for specific materials, systems and methods

We require information from authors about some types of materials, experimental systems and methods used in many studies. Here, indicate whether each material, system or method listed is relevant to your study. If you are not sure if a list item applies to your research, read the appropriate section before selecting a response.

Materials & experimental systems

n/a	Involved in the study
☒	☐ Antibodies
☒	☐ Eukaryotic cell lines
☒	☐ Palaeontology and archaeology
☒	☐ Animals and other organisms
☐	☒ Human research participants
☒	☐ Clinical data
☒	☐ Dual use research of concern

Methods

n/a	Involved in the study
☒	☐ ChIP-seq
☒	☐ Flow cytometry
☒	☐ MRI-based neuroimaging

Human research participants

Policy information about studies involving human research participants

Population characteristics	The sample included 1st and 2nd Grade children from the Pittsburgh, PA area. In Experiment 1, participants were 60 children (Grade 1, n = 30; Grade 2, n = 30; 27 females, 24 males, and 9 children whose sex was not reported; 41.7% of the participants were White, 40.0% African American or Black, 10.0% Multi-Racial, 1.7% reported their race as Other, and 6.7% were not reported). In Experiment 2, participants were 60 children (Grade 1, n = 29; Grade 2, n = 31; 31 females, 24 males, and 5 children whose sex was not reported; 63.3% of the participants were White, 8.3% African American or Black, 15.0% Multi-Racial, 1.7% East Asian or Asian American, 1.7% South Asian or Indian American, and 10% were not reported).
Recruitment	We contacted several schools in the Pittsburgh area, and schools that agreed to partner with us advertised the study to parents. Children's parents or legal guardians then volunteered to have their child participate in the study. While the sample is not representative, self-selection biases are unlikely to influence the results because the main variable of interest (Experimental condition) was manipulated within-participants such that every child served as their own control. Every effort was made to recruit children from a diverse set of schools, including public charter schools and private schools in different neighborhoods across Pittsburgh.
Ethics oversight	The Carnegie Mellon University Institutional Review Board (IRB) approved this study. This study is approved under the the following protocol # STUDY2017_00000301

Note that full information on the approval of the study protocol must also be provided in the manuscript.
